# Online Information on Lymphedema: Systematic Review of the Quality of Online Patient Resources

**DOI:** 10.1007/s13187-025-02691-2

**Published:** 2025-08-05

**Authors:** Charlene Kok, Georgios Karamitros, Gregory A. Lamaris, Huseyin Karagoz, Michael P. Grant, Vimal Gokani

**Affiliations:** 1 Medical School, Imperial College, London, UK; 2 Division of Plastic and Reconstructive Surgery, R. Adams Cowley Shock Trauma Center, University of Maryland Medical Center, Baltimore, MD USA; 3 Department of Plastic Surgery, MedicalCenterNorth, Vanderbilt University Medical Center, 1161 21 Avenue South, D-4216, Nashville, TN 37232 USA; 4 Department of Plastic Surgery, MedicalSchool, Imperial College, London, UK

**Keywords:** Patient information, Internet, Quality, Lymphedema, Lymphedema surgery, Surgery

## Abstract

The Internet has become a major source of health-related information for patients, yet the quality of online content is unregulated, leading to the potential for misinformation and patient distress. This study aims to assess the quality of online patient information on lymphedema. A search for the term “lymphedema” was conducted on the three most popular search engines (Google, Yahoo, and Bing), identifying the first 100 relevant websites, yielding 300 websites in total. These websites were evaluated using the expanded Ensuring Quality Information for Patients (EQIP) instrument, which consists of 36 items. From 300 websites identified, 105 (35%) met the criteria for final analysis, after excluding duplicates, irrelevant sites, non-English content, sites requiring user accounts, and scientific publications targeting professionals. The median EQIP score of these sites was 22, indicating suboptimal quality based on the EQIP criteria. A significant number of sites (62.9%) did not mention surgical treatment as an option for lymphedema, and 75.2% failed to describe treatment risks. Additionally, 98.1% did not report quantitative risks. Our analysis using the EQIP tool revealed that the quality of online information about lymphedema is generally poor, largely due to the omission of key content elements such as treatment options, associated risks, and quantitative data.

**Supplementary Information:**

The online version contains supplementary material available at 10.1007/s13187-025-02691-2.

## Introduction

With the rise of the Internet as a primary source of health-related information, the quality of web-based content has become a critical factor influencing patient awareness, decision-making, and health outcomes [1]. Over 80% of Internet users now rely on the Internet for medical and health- related information, making it a significant determinant of health-seeking behaviors [2, 3]. However, the vastness of online resources often makes it challenging for patients to access accurate, objective, and reliable information [4, 5]. The consequences of inaccurate or misleading online health information can be profound, disrupting patient-physician interactions, misinforming patients, and ultimately leading to worse health outcomes [6]. These concerns underscore the urgent need for high-quality, accessible online health information.

Lymphedema, a common yet often underdiagnosed condition, exemplifies the challenges patients face in accessing reliable information. Lymphedema is a chronic condition characterized by abnormal accumulation of lymphatic fluid, leading to visible tissue swelling—typically in the arms or legs. It arises due to impaired lymphatic drainage and can cause discomfort, reduced mobility, and increased risk of infection. Despite its significant physical and psychosocial impact, lymphedema is often underrecognized or misdiagnosed [7–10]. Secondary lymphedema is particularly prevalent in developed countries, where iatrogenic causes (i.e., resulting from medical treatment such as surgery or radiation) dominate [11]. The condition is increasingly relevant as the global population ages and cancer incidence rises [12]. Lymphedema is a globally prevalent condition, estimated to affect up to 250 million people worldwide [13]. Primary lymphedema is relatively rare, with a prevalence of approximately 1 in 100,000 individuals, while secondary lymphedema is far more common and arises from causes such as oncologic treatment, trauma, or infection [13].

Recent studies reveal alarming deficiencies in patient education regarding the potential development of lymphedema following cancer treatment. For instance, only 25% of breast cancer patients reported receiving adequate preoperative information about lymphedema risk [14], while just 16.83% of gynecologic cancer patients were informed about its possibility post-treatment [15]. These gaps in awareness, combined with the high prevalence of lymphedema among cancer survivors—affecting up to one in five breast cancer patients—highlight the critical importance of reliable educational resources [16].

In cases of iatrogenic lymphedema, particularly following breast cancer therapy, plastic surgeons often play a pivotal role as the first healthcare providers to identify and diagnose the condition. This is largely due to their involvement in post-oncologic reconstruction and follow-up care, where signs of lymphedema may initially present [17, 18]. As a result, plastic surgeons are frequently the first to provide patients with information about the condition, even before they turn to online resources for further research [19]. Plastic surgeons often play an important role in lymphedema prevention and patient education, including counseling on the risk of post-surgical lymphedema, early symptom recognition, limb care strategies, and lifestyle modifications such as maintaining optimal BMI, engaging in supervised physical activity, and avoiding limb trauma or constriction. While this interaction represents a critical opportunity for patient education, it underscores the importance of ensuring that subsequent web-based information is accurate, comprehensive, and supports the clinical advice given.

Given the Internet’s widespread use and influence, ensuring the availability of high-quality online information about lymphedema is paramount. This study aims to address this critical gap by systematically evaluating the quality of online resources pertaining to lymphedema. By replicating the search behavior of a typical patient, we objectively analyzed the content of websites providing information on lymphedema, with the goal of identifying strengths, weaknesses, and areas for improvement. Our findings aim to guide efforts toward enhancing the availability of accurate, reliable, and accessible online information, ultimately empowering patients and improving outcomes.

## Methods

### Web Page Identification and Eligibility Criteria and Data Collection

We analyzed search results generated by the three most popular search engines, Google, Bing, and Yahoo. These search engines combined acount for 99% of search engine traffic in the USA and 96.23% worldwide [20]. This approach allowed us to replicate the organic Internet browsing behavior of the average internet user in order to generate the websites that would be commonly available. Search terms were selected to reflect those most frequently used by average internet users—defined here as non-specialist individuals seeking online health information—based on search engine market share data compiled by “Search Engine Market Share Unite Worldwide” (accessed on August 14, 2024) [20]. An advanced web search was conducted using the terms “lymphoedema” and “lymphedema” across search engines to identify websites containing relevant information. These were the only terms used, as there are no widely recognized synonyms for lymphedema in the medical literature. The Internet search was conducted in an incognito browser mode, with cookies and browsing history cleared, and the location feature disabled, to ensure that the results were reproducible, reliable, and unbiased. The first 100 websites from each search engine were included in the study as it was reasoned that most individuals looking to find out more about lymphedema would limit their searches within the first 100 results [5, 21–24].

Two reviewers (GK and CK) independently evaluated each website using the expanded EQIP tool. Any discrepancies in scoring were resolved through a structured consensus process involving joint review and discussion until agreement was reached [25]. This approach is consistent with established methods used in content validation and expert panel agreement [25]. Our sample com- prised initially from 300 websites in total. Of the 300 web pages, duplicates were removed before they were screened, and further websites were removed using the following exclusion criteria: (1) websites not relevant to the subject of lymphedema, (2) websites promoting lymphedema services or products without explaining the condition, and (3) websites with information not intended for patients, such as that of scientific journals intended for healthcare personnel and researchers, as it was assumed that patients would not have access to the full papers and limited understanding of the specialized scientific content of the manuscripts. After applying the exclusion criteria, 105 websites remained and were subjected to the evaluation and assessment process with the EQIP tool.

The 105 websites, comprising the data sample of our analysis, were classified into the following categories: (1) hospital, (2) practitioner, (3) professional society, (4) charity/non-profit organization, (5) news service, (6) encyclopedia, (7) industry, (8) academic centre/educational institution, and (9) patients groups as performed in previous studies evaluating web-based health information [5, 21–23].

## Data Assessment Tool: EQIP Instrument

The 105 websites that fulfilled the eligibility criteria were then screened and evaluated using the modified EQIP tool [24, 26]. The EQIP tool consists of a 36-item checklist is designed to critically evaluate documents, leaflets, or websites providing medical information based on three classifications: (1) content data (items 10–18), (2) identification data (items 19–24), and (3) structure data (items 25–36) (Table S1). Items 1–9 assess basic accessibility, general formatting, and document suitability. Upon completion of this assessment, it was revealed that the inter-rater reliability of the modified EQIP tool was impeccable (kappa coefficient=0.84), while the inter-class correlation was 95% [26, 27].

The EQIP instrument has been utilized in previous studies evaluating the quality of online health-related information [5, 21–23, 28–35]. In our study, we used the updated EQIP tool, which operates on a binary scale with responses of “yes,” “no,” or “not applicable” (indicating no score) [21, 36].

## Items Describing Treatment Options for Lymphedema

Items 4, 5, and 6 of the EQIP tool are designed to evaluate whether webpages explained treatment options for lymphedema. The first-line treatment for moderate lymphedema typically involves various forms of compression therapy. For more severe or refractory cases, microsurgical techniques such as lymphovenous bypass or lymphaticovenular anastomosis may be used. These procedures involve connecting small lymphatic vessels directly to nearby veins to help drain excess lymphatic fluid and reduce swelling [37, 38]. Items 4, 5, and 6 were specifically used to differentiate if the websites provide information on surgery as a potential treatment option for lymphedema and whether such treatments were explained or elaborated on. Specifically, item 4 would critically assess whether the websites adequately describe the purpose of treatment procedures. Item 6 assesses whether the websites provide information on the surgery being a potential treatment for lymphedema, while item 5 assesses whether the websites provide information on treatment alternatives other than surgery. This allowed us to infer if surgical treatment is communicated as a potential treatment option in the information provided for patients online.

## Morbidity and Mortality Risks

Items 9 and 10 of the updated EQIP tool were created to assess the risks of surgical interventions for lymphedema (Table S1). Item 9, in particular, was used to critically assess whether the webpages sufficiently refer to the quantitative risks and complications related to the surgical procedure of lymphovenous bypass or lymphaticovenular anastomosis (Item 9, Table S1) [38]. Conversely, item 10 was used to evaluate whether the webpages referred to the quantitative risk of each complication of the lymphovenous bypass or lymphaticovenicular anastomosis surgery as a percentage (item 10, Table S1).

## Statistical Analysis

Statistical analysis was performed using R version 3.3.2 (R Core Team, GNU GPL v2 License), R Studio version 1.0.44 (RStudio, Inc. GNU Affero General Public License v3, Boston, MA, 2016) with the graphical user interface (GUI) rBiostatistics.com alpha version (rBiostatistics.com, London, UK, 2017). For categorical variables, we provide both the frequency in count and the corresponding percentage for each value. Pearson’s *χ*^2^ test was used to compare categorical variables. The Student’s *t*-test was used for continuous variables. The level of statistical significance was set at a *p*-value of ≤ 0.05; all the tests were two-sided. The scoring system assigned equal weight to all items, allowing each webpage to achieve a maximum score of 36 and a minimum score of 0. Subsequently, an aggregate score was computed for each website. High-score web pages were defined as those with an EQIP score above the 75th percentile, while those below the 75th percentile were defined as low-score web pages. This classification has been proposed by previous studies conducted by Karamitros et al. [5, 21–23]

## Results

### Websites Presenting Information on Lymphedema

**Fig. 1:**
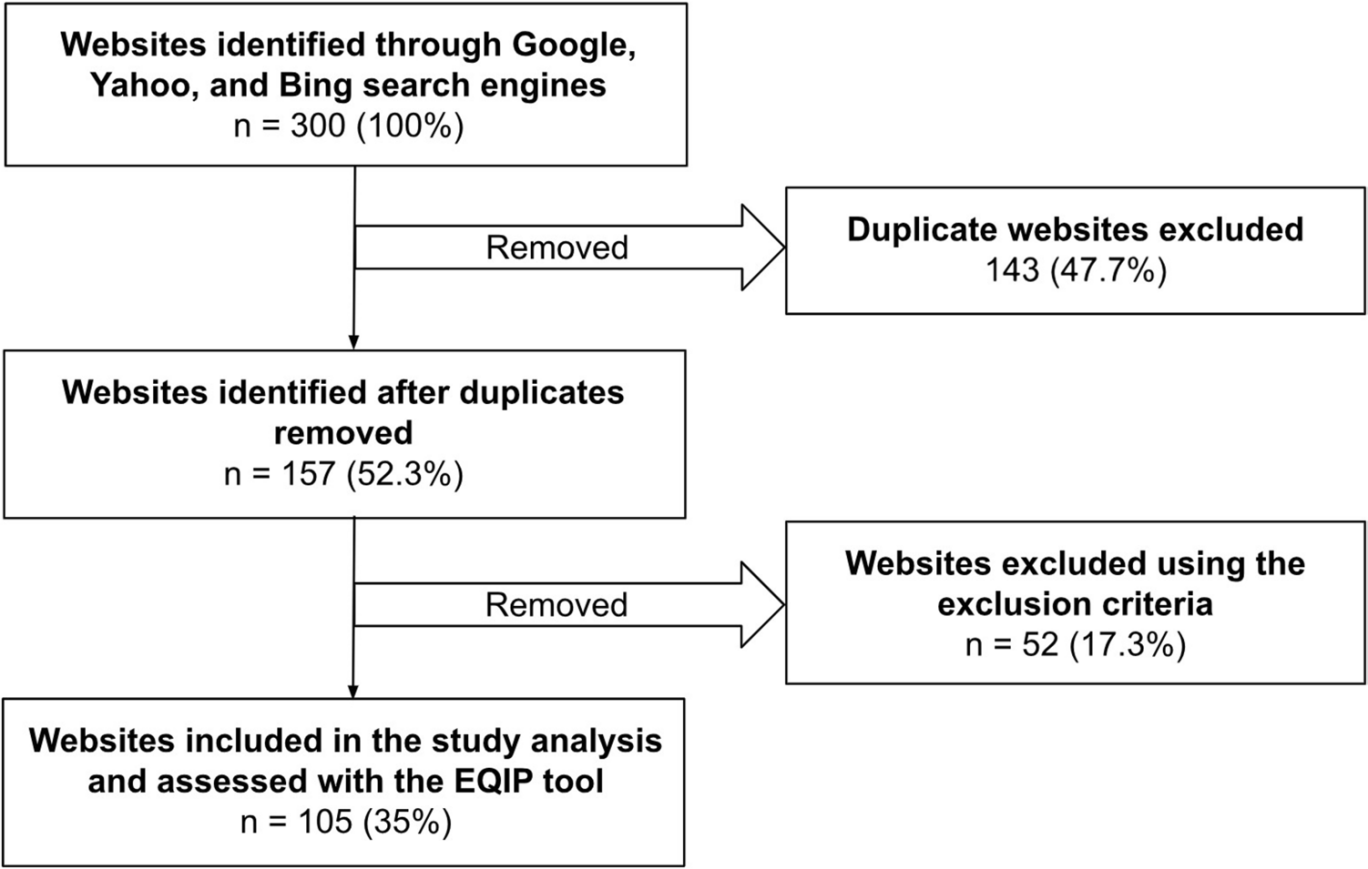
Flowchart of website selection for analysis

Three hundred websites containing a combination of the keywords “lymphoedema” and “lymphedema” were found using Google, Yahoo, and Bing Internet search engines. After the removal of duplicates, non-English websites, irrelevant websites, and websites targeting a scientific audience, 105 websites remained for qualitative and quantitative assessment with the modified EQIP instrument (Figure 1).

Of the 105 websites, 30 (28.6%) were provided by hospitals, 18 (17.1%) by practitioners, 17 (16.2%) by professional societies, 14 (13.3%) by charity/non-profit organizations, 8 (7.6%) by news services, 6 (5.7%) by academic centres, 5 (4.8%) by encyclopedias, 4 (3.8%) by industry, and 3 (2.9%) by patient groups (Figure S1).

## Characteristics of the Sample Sorted by Country of Origin

Websites are categorized by country of origin in Figure S2. Most of the websites (70; 65.7%) were from the UK, followed by 17 (16.2%) from Australia, 10 (9.5%) from the USA, 4 (3.8%) from New Zealand, 2 (1.9%) from Singapore, 2 (1.9%) from Ireland, and 1 (1.0%) from Germany.

## Scoring of the Websites Assessed

The median EQIP score was 22 (inter-quartile range 19-25) (Figure S3). Websites that scored above the 75th percentile, i.e., scoring ≥25 were described as high-score websites. Based on this assessment, 22 (21.0%) websites were determined to be high-score sites, and the remaining 83 (79.0%) were determined to be low-score sites.

**Fig. 2:**
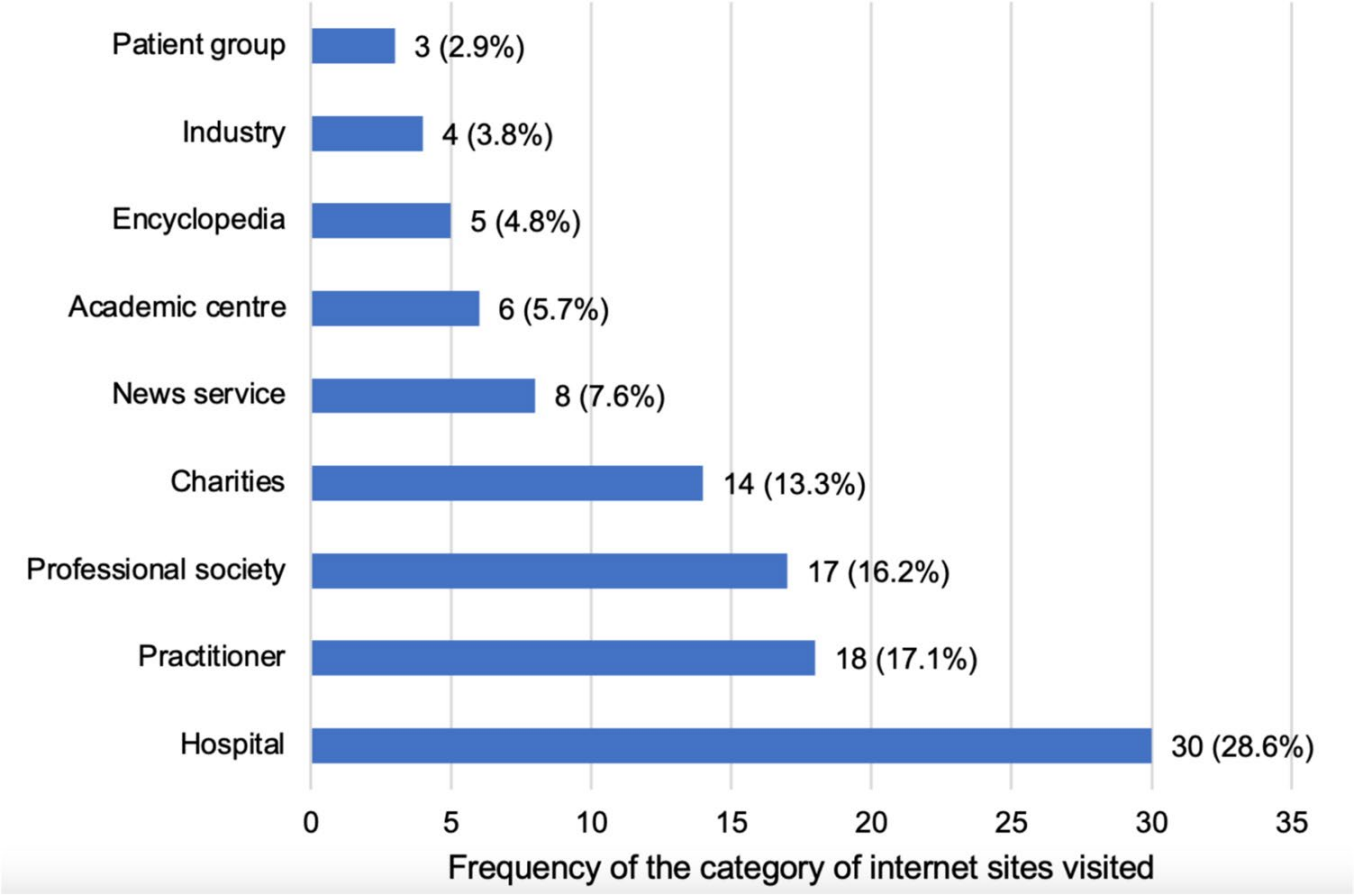
EQIP score distribution by website category. Notes: The boxplot illustrates the EQIP score distribution across different categories of websites analyzed in the study. Charities and patient group websites achieved the highest median scores, reflecting better quality and readability. Conversely, academic center and hospital websites demonstrated wider variability, with some scoring lower than the median. Categories such as encyclopedias and news services exhibited more consistent, moderate-quality scores. These findings highlight significant variability in the quality of lymphedema-related information depending on the website category

**Fig. 3:**
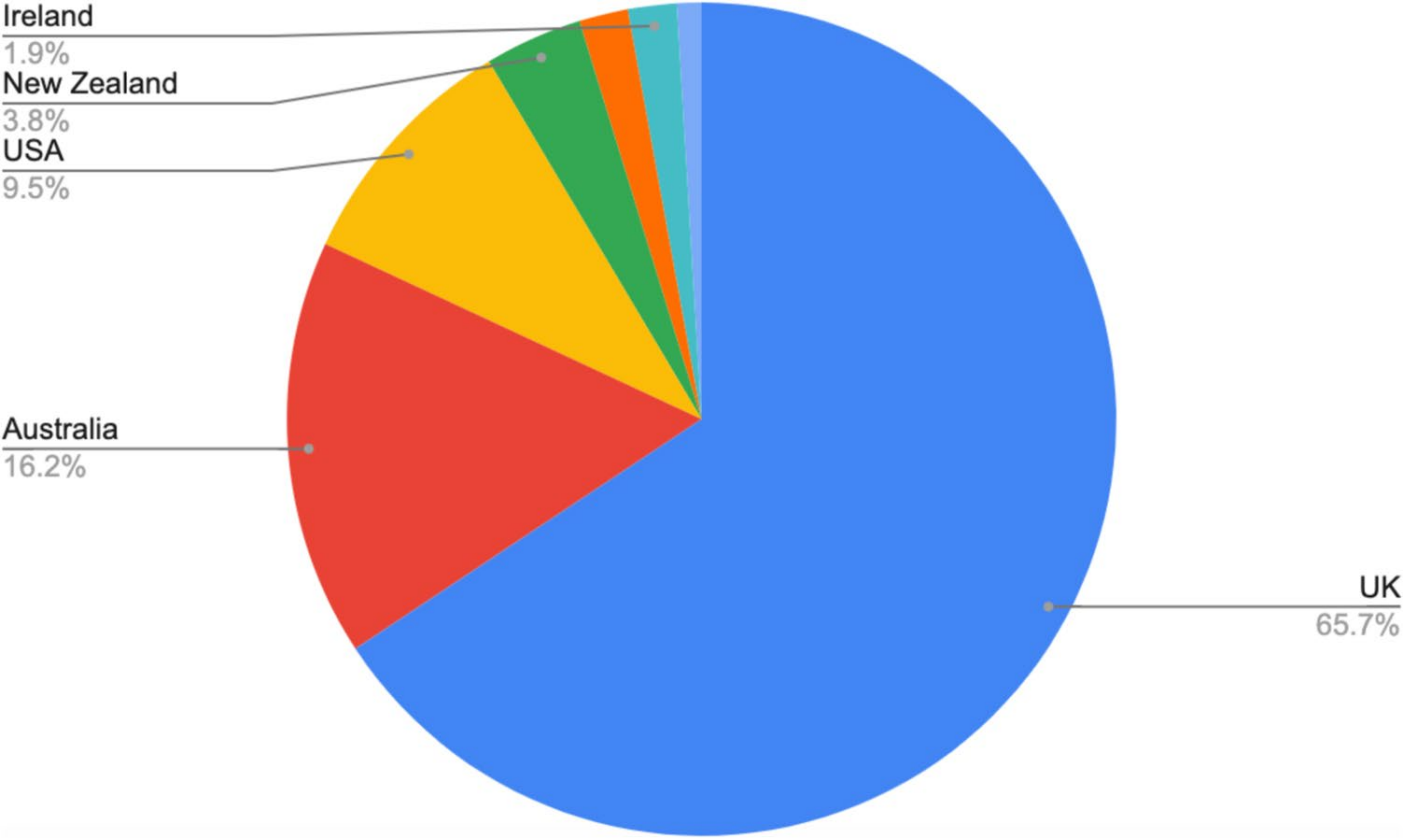
EQIP scores by website country of origin. Notes: The boxplot shows the distribution of EQIP scores across websites grouped by their country of origin. Websites from the USA demonstrated the widest score variability, with several achieving high-quality scores. Websites from the UK exhibited a higher median score compared to other countries but also displayed notable variability. Australian websites showed a relatively balanced distribution, while sites from Ireland, New Zealand, and Singapore had narrower score ranges, reflecting consistent but moderate quality. The thick horizontal lines represent the medians, while the ends of the whiskers show the minimum and maximum scores

Regarding the source of information, websites created by patient groups and charities demonstrated higher scores compared to those developed by hospitals or medical practitioners (Figure 2). While this difference did not reach statistical significance (*p* = 0.25843), the trend is notable and suggests that patient-centered organizations may produce more accessible or comprehensively structured resources. This finding highlights a potential strength of the current study in detecting qualitative differences among information providers that may hold clinical and communicative relevance, especially for patient engagement and health literacy strategies. Similarly, a subgroup analysis based on the country of origin revealed that websites from the US had the highest median scores compared to those from the UK, Ireland, and Australia (Figure 3). Yet, this variation was also not statistically significant (*p* = 0.5855).

## Websites Attaining the Top Score

The five top-scoring websites all had a score above the 97th percentile (EQIP≥29). The website with the highest score obtained 30 points on the 36-point scale. Three of the five top-scoring websites were developed in the UK, and two were designed in Australia (Table S2). Of the 70 websites from the UK, 14 (20%) obtained high EQIP scores (22 or higher). This represented more than half of the total (*n* = 22) high-score websites. Upon closer analysis, these top-scoring websites were characterized by well-organized content with clear headings, defined authorship and contact information, language tailored to lay audiences, and transparency about content sources and review dates. These features likely contributed to their superior EQIP performance and may represent important benchmarks for the development of future high-quality online patient resources.

## Discussion

In this comprehensive systematic review, we evaluate the Internet’s role as a critical resource for patient education on lymphedema. Our analysis revealed both positive and negative findings. Key negative findings included the following: (i) the majority of websites offered substandard educational content, and (ii) many sites failed to adequately address the risks associated with treatment options. A neutral observation was (iii) the considerable variability in the overall quality of available resources. One notable positive finding was (iv) the generally well-structured and navigable organization of most websites. While the Internet continues to serve as a pivotal platform for patient education, these quality gaps highlight an urgent need for the development of more reliable, comprehensive, and patient-centered lymphedema resources.

While patient-physician consultations are the cornerstone of medical information, the Internet is often the first resource patients consult, particularly in plastic and reconstructive surgery, where many patients research their options online [2, 5, 21, 39–41]. Ensuring the accuracy, readability, and comprehensibility of online content is critical, as poor health literacy and low-quality information exacerbate health disparities, hinder patient satisfaction, and negatively impact outcomes [6, 42].

Well-informed patients are better equipped to make decisions and collaborate effectively with healthcare providers, leading to improved outcomes. However, the unregulated nature of the Internet facilitates the proliferation of low-quality medical information, potentially undermining patient adherence and outcomes. Our study provides a novel evaluation of 300 lymphedema websites using the modified EQIP tool. To our knowledge, no prior research has specifically assessed the quality of online content for lymphedema. Our findings are consistent with prior studies on other medical topics [5, 21, 28, 30, 43, 44], with a median EQIP score of 22 (61.1% of the maximum). While website structure was rated highest, content quality scored the lowest.

A significant gap was identified in the discussion of treatment risks. Although treatment options were often mentioned, 62.9% of websites failed to address surgical interventions for lymphedema, and 76% did not include any risk information, with 98.1% omitting quantitative data. This omission was particularly prevalent on private medical practitioner websites, which may avoid discussing risks to attract patients [21, 43]. While compression therapy remains the first-line treatment for lymphedema, advancements in surgical techniques—such as lymphovenous bypass, lymph node transfer, and microsurgical reconstruction—have significantly improved outcomes [37, 38, 45–49]. Comprehensive and transparent information on treatment risks is vital to ensure informed decision-making and patient engagement [38, 50, 51].

Readability was another concern, as most websites exceeded the recommended sixth-grade reading level. Although 86.4% of sites used short sentences, only 49.3% included relevant figures or graphs, and 45.7% personally addressed the reader. Simplifying medical language is essential to enhance understanding and engagement [5, 21, 44, 52].

Interestingly, websites by patient groups had the highest median EQIP scores, contrasting with previous studies where hospital and academic websites performed best [5, 21]. This discrepancy may reflect inconsistencies in how lymphedema is defined and detected clinically [9, 53]. The absence of a universally accepted definition has likely contributed to suboptimal educational content.

While this study focused primarily on English-language websites from countries such as the US and UK, the patterns observed—particularly the variability in content quality and the underrepresentation of critical risk information—highlight challenges likely relevant across diverse international contexts [41]. Accordingly, healthcare organizations worldwide should prioritize the development of high-quality, culturally and linguistically tailored online resources to meet the educational needs of broader patient populations. Incorporating cultural, linguistic, and educational diversity into web design can improve global patient engagement. An external “stamp of approval” for reliable websites could further guide patients toward trustworthy resources.

Empowering patients with high-quality information is essential for enhancing their role in healthcare decision-making. When patients are well-informed, they collaborate more effectively with providers, leading to better communication, adherence, and outcomes [41, 44, 54]. Addressing the deficiencies in online lymphedema resources is an important step toward equitable, patient-centered care.

## Limitations

This research design has certain limitations. We began with an exploratory phase in which a combination of commonly used keywords was tested to simulate the likely search behavior of internet users seeking information on lymphedema. This initial approach was used to inform the construction of our formal search strategy, which is detailed in the “Methods” section and was applied systematically for the final website selection and evaluation. However, Internet search behaviors can vary, so while we designed a comprehensive search strategy, some relevant websites may not have been included in our initial data collection. To account for the diversity in user-specific search strategies, we made efforts to compile an exhaustive list of websites that patients might encounter online.

Our sampling strategy, which included the first 100 search results from each of the three most commonly used search engines, may have excluded lower-ranked websites that are less search- engine optimized but potentially still of high quality. This introduces a potential selection bias that could affect generalizability. However, it is a commonly accepted practice in similar content quality studies to limit the analysis to the first 100 search results or fewer, as this reflects actual user behavior [5, 21–23, 28, 30, 43]. Most Internet users are unlikely to navigate beyond the first few pages of search results and virtually never beyond the first 100 links. Therefore, while we acknowledge this limitation, we believe that our methodology approximates real-world information-seeking behavior and provides ecologically valid insights into the quality of online patient education on lymphedema. Additionally, language barriers present a limitation; we only evaluated websites that provided information in English, which restricted our insight into web pages offering content in other languages. However, English is widely spoken globally and serves as the first or second language in many countries [55], making it reasonable to assume that our study captures the majority of relevant web pages. Future research could expand by critically assessing the quality of websites in languages other than English.

Moreover, our analysis employed the EQIP instrument, a validated tool for content evaluation, though it was not specifically designed to assess lymphedema-related information. It is important to acknowledge that both the original EQIP (2004) and the expanded EQIP (2008) were developed over a decade ago. As such, they may not fully capture emerging trends in how health information is consumed online today—such as interactive web content, multimedia platforms, and social media channels. Nonetheless, the expanded EQIP remains one of the most widely used and reliable instruments for assessing the structural and informational quality of online health resources across medical domains [5, 21–23, 28, 30, 43].

Finally, the ever-changing nature of the web presents another limitation. New information is added daily, and although we used an incognito search strategy to minimize location-specific biases, we acknowledge that the websites and results could differ based on individual user settings. Additionally, websites may undergo internal updates and revisions. As a result, our analysis offers a snapshot of the information patients might encounter at a given time. Despite these limitations, we believe our study is representative of the web-based information that patients seeking lymphedema-related content may encounter.

## Conclusion

Our comparative analysis highlights that the low quality, structure, and readability of online information contribute to disparities in patient access to lymphedema care. While some websites scored well on the EQIP tool, none fully met all checklist criteria. Improving the quality of online information could help reduce barriers to surgical care. In the meantime, it is the responsibility of physicians and surgeons to provide or guide lymphedema patients to reliable resources and ensure they receive adequate preoperative counseling. Future research should incorporate direct input from patients and patient educators to ensure that online materials are aligned with user needs. Additionally, expanding such evaluations to include non-English and globally diverse websites is essential for addressing broader disparities in digital health equity and accessibility.

## Supplementary Information

Below is the link to the electronic supplementary material.

ESM 1 DOCX (13.2 KB)

## Data Availability

Data are available upon an official request to the corresponding author.
