# Supplementary material for: Online Information on Lymphedema: Systematic Review of the Quality of Online Patient Resources
Source: J Cancer Educ. 2025 Aug 5;41(3):536–44. doi: 10.1007/s13187-025-02691-2 (PMC13222276; doi:10.1007/s13187-025-02691-2)
Supplement: Supplementary file 1 — DOCX (13.2 KB) [file 13187_2025_2691_MOESM1_ESM.docx]

# Supplementary Appendix

## Supplementary Figure Legends

**Figure S1:** Frequency of Categories of Internet Sites Evaluated in the Study.

**Figure S2:** Geographic Distribution of Websites Evaluated in the Study.

**Figure S3:** Distribution of EQIP Scores Across Evaluated Websites.

*Notes: This histogram depicts the distribution of EQIP scores (x-axis) for the 105 websites included in the analysis. The scores ranged from 0 to 36, with a median score of 22 (standard deviation: 3.955). The line represents a normal distribution fitted to the data. Dark-shaded bins indicate websites scoring above the 75th percentile (EQIP ≥ 25), reflecting the highest-performing websites in terms of quality, accuracy, and readability.*

## Supplementary Tables Legends

**Table S1:** Evaluation of Website Content Based on the EQIP Tool Criteria.

**Table S2:** Top-Ranked Websites Based on EQIP Scores.
